# Decline in Memory, Visuospatial Ability, and Crystalized Cognitive Abilities in Older Adults: Normative Aging or Terminal Decline?

**DOI:** 10.1155/2017/6210105

**Published:** 2017-05-29

**Authors:** R. Bendayan, A. M. Piccinin, S. M. Hofer, D. Cadar, B. Johansson, G. Muniz-Terrera

**Affiliations:** ^1^MRC Unit for Lifelong Health and Ageing, University College of London, Faculty of Population Health Sciences, London, UK; ^2^Department of Psychology, University of Victoria, Victoria, BC, Canada; ^3^Department of Psychology, University of Göteborg, Göteborg, Sweden

## Abstract

The aim of this study is to explore the pattern of change in multiple measures of cognitive abilities in a sample of oldest-old adults, comparing two different time metrics (chronological age and time to death) and therefore examining both underlying conceptual assumptions (age-related change and terminal decline). Moreover, the association with individual characteristics as sex, education, and dementia diagnosis was also examined. Measures of cognitive status (Mini-Mental State Examination and the Swedish Clock Test) and tests of crystallized (knowledge and synonyms), memory (verbal memory, nonverbal long-term memory, recognition and correspondence, and short-term memory), and visuospatial ability were included. The sample consisted of 671 older Swedish adult participants of the OCTO Twin Study. Linear mixed models with random coefficients were used to analyse change patterns and BIC indexes were used to compare models. Results showed that the time to death model was the best option in analyses of change in all the cognitive measures considered (except for the Information Test). A significant cognitive decline over time was found for all variables. Individuals diagnosed with dementia had lower scores at the study entrance and a faster decline. More educated individuals performed better in all the measures of cognition at study entry than those with poorer education, but no differences were found in the rate of change. Differences were found in age, sex, or time to death at baseline across the different measures. These results support the terminal decline hypothesis when compared to models assuming that cognitive changes are driven by normative aging processes.

## 1. Introduction

Increasing levels of population aging turns research about aging related cognitive decline into a worldwide priority. Although it is well known that cognitive abilities decline with age, a better understanding of the underlying processes and individual differences in cognitive aging remains a main objective in current cognitive aging research [1].

Cognitive decline in aging studies is often explored using linear mixed models [2]. These models consist of two levels of analysis that allow researchers to explore how individuals change over time and how these changes vary across individuals [3, 4]. Depending on the context, time can represent any appropriate scale, ranging from milliseconds to millennia, and can be centered for all individuals to represent distance from any event: birth (i.e., age), time since baseline measurement, time to diagnosis, or time to death. While time is a generic concept that passes (within a person) in the same way, regardless of how it is specified [5], different time metric specifications imply a specific alignment of the individuals' scores relative to other people in the sample. From a methodological perspective, it has been shown that if scores are aligned according to a time metric that does not reflect the progression of the processes driving change, the measurement model is misidentified and estimation of the parameters of change is distorted [6].

Moreover, choosing the most appropriate time metric to model cognitive change is not only a statistical issue, but also a theoretical one. Different time metrics assume that the changes are driven by different underlying processes. For example, selecting age as time metric assumes that all the individuals at each age are in the same stage of the causal process (aging) that is driving the cognitive changes. Therefore, when changes are modelled using chronological age, these changes are considered as age-related changes, which are often conceptualized as normative aging changes [7]. However, cognitive changes in older adults may not be driven by age-related changes but by their proximity to death, proposed by the terminal decline hypothesis. This hypothesis was first described by Kleemeier [8] and extended by K. F. Riegel and R. M. Riegel [9] who hypothesized that approximately 5 years prior to death there is an acceleration in cognitive decline. Since then, a great body of research has examined this hypothesis in older adults (for a review see [10, 11]) and several studies have modelled change in cognitive abilities assuming time to death as time metric [12–14]. On the other hand, a number of reports which have modelled change in cognitive abilities in older adults assumed this change as a function of chronological age (e.g., [15, 16]).

Most of the studies explored measures of cognitive abilities using only one time metric making an a priori assumption to conceptualize the change in these abilities (e.g., [12, 17]). Johansson et al. [12] explored cognitive change in older adults using only the time to death model and found that proximity to death is predictive of decline in indicators of crystallized knowledge and verbal abilities. Laukka et al. [17] compared different cognitive measures assuming a terminal decline model and also found that some tasks are more sensitive to terminal decline than others. Moreover, they highlighted the need of further research to distinguish whether there is a differential effect for tasks measuring crystallized cognitive abilities compared to other tasks measuring, for example, fluid abilities. Few studies compared models with different time metrics [18–21]. Gerstorf et al. [18] explored change in cognitive function (using a Digit Letter test) and compared models using chronological age and time to death as time metrics. They found that the time to death metric explained better the variability of this cognitive measure than the chronological age metric, suggesting that changes in performance-based variables, as cognition, were primarily driven by mortality-related processes. Sliwinski et al. [20] focused on memory loss and compared different time metrics as chronological age, time to dropout, and time to dementia diagnosis. These authors considered that one possible explanation of the attrition effects is that individuals tended to drop out from the study as a result of death, and therefore they included time to death as time metric when studying individuals free of dementia. They concluded that chronological age may not be the best time metric to describe change in cognitive abilities in older adults and that time to an event (e.g., death or dementia diagnosis) models may be more useful to describe the pattern of change in cognition in older adults. Similar results were found by Ram et al. [19] who examined memory changes across models that aligned scores according to different time metrics, as chronological age, time to disability, and time to death, in a sample of deceased and disabled individuals. Thorvaldsson et al. [21] explored change in perceptual speed as a function of age and time to death, finding time to death as the best metric. In sum, evidence about differential patterns of change in multiple measures according to contrasting metrics of time is scarce.

The aim of this study is to explore the pattern of change in measures of cognitive status and specific measures of cognitive abilities in a sample of oldest-old adults, comparing two different time metrics (chronological age and time to death) and therefore examining both underlying conceptual assumptions (age-related changes and terminal decline). Two brief and well-known measures of cognitive status (Mini-Mental State Examination and the Swedish Clock Test), memory (verbal memory, nonverbal long-term memory, recognition and correspondence, short-term memory), two tests of crystallized cognitive abilities (knowledge and synonyms), and visuospatial ability are included. In addition, the association with individual characteristics as sex, education, and dementia diagnosis is also examined.

## 2. Method

### 2.1. Sample

The sample consisted of 671 older Swedish adults (229 women and 442 men) that were participants of the Origins of Variance in the Old-Old (OCTO Twin Study). Further details about Swedish Twin Studies can be found in Pedersen et al. [22]. Participants were assessed on five measurement occasions at two-year intervals. At the first measurement occasion, respondents were 83.65 years old in average (SD = 3.19) and had a mean of 7.12 years of education (SD = 2.28). From this sample, 447 were never diagnosed with dementia while 224 were diagnosed with dementia at some point of the study. During and after the five waves of measurement, date of death was recorded for each participant. The same sample of deceased individuals was used in all the considered models. Only deceased individuals were included as comparing level of cognitive performance or rate of change in deceased and surviving individuals represents a comparison across individuals, rather than a focus within individuals, which is required given the conceptualization of terminal decline as a within-individual process [14, 23].

### 2.2. Measures

The OCTO Twin Study includes a number of cognitive and behavioural measures including two general measures of cognitive status, four measures of memory, two of crystallized abilities, and one of visuospatial abilities. We included standard measures to screen dementia in clinical settings [24], for example, the MMSE, based on their relevance and extended use to identify individuals in studies examining the association between cognitive impairment and mortality. Memory tasks, in general, and verbal memory tasks, in particular, were included as these have shown to be one of the strongest predictors of dementia risk in older adults [25, 26]. In addition, research has highlighted the importance of examining measures of visuospatial and crystallized cognitive abilities not yet included in previous studies with similar aims [11, 17].

### 2.3. Cognitive Status

Cognitive status was measured using the Mini-Mental State Examination (MMSE [27]) and the Swedish Clock Test [28]. The MMSE consists of 21 questions on orientation, immediate and delayed recall, naming, spelling, and simple arithmetic and constructional praxis. The Swedish Clock Test, which is similar to the Clock Drawing Test, includes three subtasks: clock drawing and setting hands to a certain time; setting the hands of a wooden clock with no numbers on the face to certain standard times; and reading the clock. Greater values indicate a better cognitive status.

### 2.4. Memory

Verbal memory was assessed by a Prose Recall Test [29]. This test is a Swedish language Prose Recall task similar to those from the Wechsler Memory Test [30]. Respondents were asked for immediate free recall of a brief story. Higher scores are indicative of a greater level of verbal memory.

Nonverbal long-term memory was assessed with the Thurstone Picture Memory Test [31]. Respondents were shown 28 pictures and asked for recognition of these among three lures. Higher scores indicate greater levels of nonverbal memory.

Memory recognition and memory correspondence were assessed by the two subtests of the Memory in Reality Test (Johansson, 1988/89). This test was developed as an alternative to standard list-learning tasks and asked the participants to place real-life objects at the same location as they were previously placed. Higher scores are indicative of better memory.

Short-term memory was assessed by the Digit Span Forward and Backward Test (WAIS [32]). The participants were asked to recall orally presented digits in the same and reverse order as they were presented. Higher scores represent higher levels of short-term memory.

### 2.5. Visuospatial Ability

Visuospatial ability was assessed with the Kohs Block Design Test [33]. Respondents were asked to reproduce with blocks a pattern shown in cards. Higher scores are indicative of a greater level of visuospatial ability.

### 2.6. Crystallized Abilities

These abilities can be described as the knowledge and skills acquired through education and other cultural experiences over the life span [34, 35]. In the OCTO Twin Study, two tests from the Swedish version of the Wechsler Adult Intelligence Scale (WAIS [32, 36]) were used to assess crystallized knowledge: the Swedish version of the Information Task and the Synonyms Test. The Information Task includes questions of general knowledge, which requires participants to provide answers to questions assessing acquired semantic knowledge of facts. The Synonyms Test is a verbal meaning test where the respondent has to find a synonym that matches a target word among five alternatives. Higher scores represent higher levels of knowledge.

### 2.7. Statistical Analyses

Linear mixed models, with random intercepts and slopes, were estimated using SAS Proc Mixed [3, 37], as these models allow capturing between- and within-individual variability. Conditional models for chronological age and time to death were fitted to each cognitive measure, adjusted for baseline age, sex, education, dementia diagnosis at any time point across the five measurement occasions, and time to death at baseline. Age was grand-mean centered and time to death was centered at two years before death (to avoid placing the intercept at a point in time when the measurement of cognition is not possible). Dichotomous covariates were effect-coded and continuous variables were centered. Baseline age was centered at 85 years, education at 9 years, and time to death at baseline at 5 years before death. To separate between-individual changes from within-individual changes in each model, baseline age and time to death from baseline were included in the models [6, 23, 38]. For each temporal index we also explored models describing change at a constant (linear trajectory) and changing rate (quadratic trajectory). That is, for each cognitive measure, four models were compared: (1) chronological aging linear change, (2) chronological aging quadratic change, (3) time to death linear change, and (4) time to death quadratic change. More detailed information and equations are shown in the Appendix. The Bayesian Information Criterion (BIC [39]), an index that combines information about model goodness of fit and parsimony, was used to compare the fit of the models. Models with lowest BIC are preferred and differences between models were compared based on Raftery's (1995) proposed criterion. Raftery (1995) reviewed model selection criterions using BIC index and proposed ranges of differences between BICs of nested models as grades of evidence of these differences, which were categorized as weak (0–2), positive (2–6), strong (6–10), and very strong (>10). We also calculated the proportional reduction in residual variance from unconditional means models, which has been conceptualized as a ΔPseudo *R*^2^ [40]. When examining the association with individual characteristics variables, only significant parameter estimates were reported in order to facilitate the interpretation of the results.

## 3. Results

### 3.1. Time Metrics

For each cognitive measure, the BIC indices for the four models were compared. As can be seen in Table 1, for measures of cognitive status, quadratic time to death models fitted the data best. With regard to memory and visuospatial ability, linear time to death models were also the best fitting models. However, when crystallized abilities were explored, time to death was the model that provided a better fit for the Synonyms Test and the chronological age model was the best for Information Test. According Raftery's (1995) grades and considering the sample size of our study, the evidence of the differences between the models was very strong in all the cognitive measures. With regard to the random effects (Table 2), we found that in all the measures there was significant variability at intercept level but only significant variability at slope level was only found for the Prose Recall and Block Design Tests and crystallized cognitive abilities.

### 3.2. Predictors of Change

Table 2 provides the significant parameter estimates for the intercept and slopes and the covariates associations identified for the best fitting models. On the one hand, a common pattern was found across all cognitive measures: (a) a significant decline over time; (b) dementia diagnosis which was negatively associated with the level and the slope; and (c) education which was positively associated with level but not with slope. On the other hand, some differences were found in age, sex, or time to death associations with the intercept and slope across the different measures of cognitive function.

With regard to baseline age, results showed that people older at study entry had lower initial scores on the MMSE and the Clock Test but only showed a faster decline in MMSE over time. For the memory tasks and visuospatial ability, older people also performed worse in the Memory Recognition, Memory Correspondence, and Block Design Tests, but age differences in rate of decline were only found for Digit Span. With respect to the crystalized abilities, there were no age differences at intercept or slope level.

Significant sex differences were found for Memory Correspondence and the Information Tests. Women performed better than men on Memory Correspondence but worse on the Information Test. No sex differences were found on the slope for any of the tests examined, except for the Information Test.

Time to death at the baseline was significantly positively associated with the level of MMSE, Clock, Memory Correspondence, and Digit Span Test and negatively associated with Prose Recall and the Information Test. Individuals who agreed to participate in the study when they were closer to death had higher cognitive scores than those who were further from death. Significant slope differences were found for all these variables, where individuals closer to death at study entry declined faster than individuals who entered the study further from death.

## 4. Discussion

The aim of this study was to explore the pattern of change in cognitive status and specific measures of cognitive abilities in a sample of oldest-old adults, comparing two different time metrics (chronological age and time to death) and therefore examining both underlying conceptual assumptions (age-related changes and terminal decline). Moreover, the association with individual characteristics as sex, education, and dementia diagnosis was also examined.

With respect to the time metrics, results showed that the time to death model provided a better representation of change for cognitive status, memory, and visuospatial ability. However, the results in the change in crystallized abilities did not follow such a consistent pattern. Although the trajectories of the scores in the Synonyms Test were better explained by a time to death model, the model that better described change in the Information Test was chronological age. Overall our results are consistent with those studies supporting the terminal decline hypothesis [11–13, 17, 18] and also provide some support to the distinction of the processes associated with decline in crystallized and other abilities suggested by previous research [11, 17]. Specifically, our findings suggest that in these oldest-old adults the changes in cognitive abilities can be better conceptualized within the framework of the terminal decline hypothesis and that there could be different underlying processes driving decline in some crystallized abilities. However, there is a still a need for further research to address which other processes might be underlying decline in other measures of crystallized abilities not considered here. In addition, it should be noted that terminal decline is part of normative aging and it is exceptionally complex to disentangle age-related changes, conceptualized as normative aging, and mortality-related changes, conceptualized as terminal decline. This complexity has been already discussed in previous research [18] (Steinerman et al., 2011) and future studies with cognitive data in previous developmental stages are needed to capture these complex interrelationships.

With respect to the predictors of change for each cognitive measure fitted to the time metric that provided the best fit, results showed a common pattern in all the studied variables. First, a significant decline in cognitive abilities over time was found regardless of the time metric used, consistent with previous research [19, 20]. Second, individuals diagnosed with dementia had lower scores in all the measures of cognition and showed a faster decline compared to individuals not diagnosed with dementia, also consistent with previous research [13, 20]. Third, more educated individuals performed better in all the measures of cognition than those with poorer education, but no differences in the rate of change by education were found. These findings were partially consistent with previous research. Education is generally significantly associated with level of performance but its association with rate of decline is not clear. Some studies have found an association only in some ranges of ages [41] or participants characteristics, for example, dementia [42]. A possible explanation could be found in the work of Muniz-Terrera et al. [13] which highlighted the differences in this association before and after the onset of terminal decline; however, further research is needed to address this possibility. Laukka et al. [17], within a single study, found that education was only related to rate of change for visuospatial ability. The variability of the findings about education and rate of decline in cognitive function could be due to the variations in study design or the different measures of education, as Anstey and Christensen [43] suggest, or the methods of analysis, as suggested by Piccinin et al. [42]. However, further research that integrates results from different studies using different measures of cognition and different measures of education should deepen our understanding of the association between education and rate of decline in cognitive function.

Associations with age, sex, and time to death differed across measures of cognitive abilities. On average, older people had poorer performance but baseline age associated differences in speed of decline were only found for MMSE and short-term memory. These results are partially consistent with previous research [12, 18, 42] and provide some evidence of the distinction between cognitive status measures and more domain-specific ones, which might have interesting implications from a psychometric point of view. With regard to sex, only intercept differences were found for Memory Correspondence and the Information Test. Women had better performance than men in the Memory Correspondence and lower scores in the Information Test on the initial level. These results are partially consistent with the findings of Johansson et al. [12] and could be explained because women have greater life expectancy [44]. Between-person proximity to death differences were found when comparing cognitive status measures and specific measures for crystallized ability, memory (except verbal memory), and visuospatial ability. A review on terminal change in cognition carried out by Bosworth and Siegler [11] highlighted that results vary depending on the measure of cognition studied. Indeed, Laukka et al. [17] indicated that further research on models including crystallized abilities measures is needed. Moreover, results showed that individuals who were closer to their death when they entered the study had a faster decline compared to those who were further from death, these results being consistent with previous studies [12, 13].

One of the strengths of our study is that it is one of the first, to our knowledge, to explore such a large number of measures of cognitive performance including measures widely used in clinical settings (MMSE and the Clock Test) and measures of memory, visuospatial, and crystallized abilities. The variety of cognitive measures considered does not only increase the comparability of our findings with previous research, which have only considered a single cognitive measure, but also enable us to address Bosworth and Siegler [11] and Laukka et al. [17] concerns on the potential variability of the results as a function of the measure of cognition examined. In addition, using linear mixed models with random intercepts and slopes allows us to capture between- and within-individual variability and to further separate between- and within-person chronology by including between-person differences in age and proximity to death at the first occasion [14, 23]. This point is especially relevant to avoid one of the potential causes for lack of convergence of the between- and within-person effects in longitudinal models of aging (for more details see [38]). Thus, the analytical strategy adopted in our study is suitable in order to meet our aim and consider terminal decline as a within-person process. However, some limitations should also be noted. First, although the sample of this study is one of the most representative of the oldest-old, these individuals may only represent a subset of survivors of their birth cohort at study entrance [45] and as they are relatively close to their death, their variability at slope level might be smaller than what we would expect from studies with younger populations and longer follow-ups. Therefore, research with different time metrics and different cognitive abilities should be carried out with younger samples. Second, in order to limit complexity of the models and obtain results that could be compared with previous research, only linear and quadratic models were examined. Further research with more measurements occasions should consider other nonlinear models as change point models, which are especially suitable models when researchers aim to estimate the point of onset of terminal decline. Third, there is an ongoing debate on the potential limitations of only including deceased individuals in these studies as the study becomes by its nature post facto and the generalization of our conclusions is limited to deceased population. Within this context, some studies have proposed using joint models for longitudinal and survival data in order to include surviving individuals in their samples (e.g., [46]). These joint models are especially appropriate to examine whether estimates of cognitive change are predictors of survival and allow the conclusions to be also generalized to surviving population. These models have not been adopted in the present study as there is still an ongoing debate on their suitability to study terminal decline as they do not specifically address Kleemeier's [8] operationalization of terminal decline [10]. It has been suggested that they mainly address a different research question as they explore whether those individuals who show decline or a faster decline compared to others show also a greater mortality risk as opposed to models that characterize cognitive decline in proximity to death and aim to examine whether those in close proximity to death are more likely to show greater cognitive decline. Further research including joint-survival models might contribute to detangling these issues.

In sum, our results support the fact that studying change according to terminal decline models might be the best option when compared to models that assume that changes are mainly driven by age-related changes [13, 17]. Moreover, our findings provide some evidence that there is a distinction between using cognitive status measures and domain-specific measures. As Steinerman et al. [7] suggested, cognitive status measures and domain-specific measures might not be equally appropriate for examining changes over time; and therefore, the type of measure used should be taken into account when choosing the time metric to model cognitive change. Future studies should replicate this one with younger populations and it would be extremely interesting to extend the present study including alternative theoretical time metrics as subjective or biological age.

## Figures and Tables

**Table 1:** BIC statistics for linear and quadratic chronological age and time to death models for each cognitive measure. These models were adjusted for baseline age, sex, education, dementia diagnosis at any time point across the five measurement occasions, and time to death at baseline.

Time metric	Trajectory shape	MMSE	CLOCK	PROSE	MEMRECOG	MEMCORRESP	DIGSPAN	BLOCK	SYNUM	INFO
Age	Linear	11739.3	9922.2	7835.2	6998.5	7160.3	6473.4	9571.9	6966.7	11529.9
	Quadratic	11739	9961.1	7881.9	7051	7210.5	6536.9	9611.5	6974.4	11516.2^*∗*^

Time to death	Linear	11662.7	9834.1	7799.2^*∗*^	6871.2^*∗*^	7122.4^*∗*^	6435.8^*∗*^	9555.6^*∗*^	6947.1^*∗*^	11549.6
	Quadratic	11589.5^*∗*^	9820.1^*∗*^	7830.7	6885.6	7161.2	6475.3	9580	6954.9	11526.3

*Note.* MMSE: Mini-Mental State Examination, CLOCK: Swedish Clock Test, PROSE: Prose Recall Test, MEMRECOG: Memory Recognition Test, MEMCORRESP: Memory Correspondence Test, DIGSPAN: Digit Span Backward Test, BLOCK: Kohs Block Design Test, SYNUM: Synonyms Test, and INFO: Information Test. ^*∗*^Indicating lowest BIC value.

**Table 2:** Significant parameter estimates for models identified as best fitting in Table 1.

	Cognitive status		Memory				Visuospatial ability	Crystallized cognitive abilities	
	MMSE	CLOCK	PROSE	MEMRECOG	MEMCORRES	DIGSPAN	BLOCK	SYNUM	INFO
Intercept	25.84 (0.39)	13.49 (0.29)	8.66 (0.36)	9.54 (0.19)	6.86 (0.20)	3.21 (0.10)	10.35 (0.52)	17.67 (0.33)	32.04 (0.84)
Age L									−0.61 (0.10)
Age Q									−0.08 (0.02)
T Death L	−1.31 (0.13)	−0.29 (0.10)	−0.22 (0.08)	−0.10 (0.04)	−0.29 (0.04)	−0.10 (0.02)	−0.55 (0.11)	−0.19 (0.04)	
T Death Q	−0.16 (0.02)	−0.04 (0.01)							
Age (baseline)	−0.36 (0.06)	−0.18 (0.04)		−0.09 (0.03)	−0.12 (0.03)		−0.30 (0.09)		
Female					0.76 (0.21)				−3.64 (0.89)
Education	0.41 (0.08)	0.16 (0.06)	0.39 (0.06)	0.08 (0.04)	0.13 (0.04)	0.13 (0.02)	0.58 (0.11)		1.84 (0.18)
Dementia	−1.34 (0.43)	−6.70 (0.31)	−5.72 (0.33)	−5.22 (0.20)	−4.58 (0.22)	−1.32 (0.11)	−6.41 (0.56)	−2.89 (0.61)	−12.83 (0.95)
T Death (baseline)	0.41 (0.06)	0.14 (0.04)	−0.32 (0.04)		0.07 (0.02)	0.03 (0.01)			−0.58 (0.11)
*Linear slope*									
Age (baseline)	−0.04 (.02)					0.008 (0.001)			0.1 (0.03)
Dementia	−1.89 (0.13)	−1.26 (0.10)	−0.59 (0.07)	−0.65 (0.03)	−0.38 (0.04)	−0.15 (0.02)	−0.47 (0.09)	−0.19 (0.09)	−1.02 (0.14)
T Death (baseline)			−0.02 (0.01)	−0.01 (0.005)	−0.01 (0.005)	−0.007 (0.003)	−0.02 (0.01)		−0.06 (0.01)
*Quadratic slope*									
Female									−0.05 (0.02)
Dementia	−0.09 (0.01)	−0.07 (0.01)							0.06 (0.02)
T Death (baseline)	−0.01 (0.002)	−0.003 (0.001)							
*Variances*									
Intercept	16.92 (1.59)	8.17 (0.64)	7.32 (0.66)	2.88 (0.24)	3.12 (0.27)	0.63 (0.07)	26.48 (2.01)	20.14 (1.66)	28.95 (5.70)
Slope linear			0.04 (0.01)				0.16 (0.04)	0.06 (0.02)	0.57 (0.12)
Slope quadratic									
Residual	11.46 (0.74)	6.43 (0.25)	5.64 (0.29)	2.35 (0.10)	3.05 (0.13)	1.41 (0.05)	10.64 (0.54)	7.45 (0.44)	15.87 (0.91)
ΔPseudo *R*^2^	.73	.23	.40	.48	.17	.08	.16	.02	.90

*Note.* MMSE: Mini-Mental State Examination, CLOCK: Swedish Clock Test, PROSE: Prose Recall Test, MEMRECOG: Memory Recognition Test, MEMCORRESP: Memory Correspondence Test, DIGSPAN: Digit Span Backward Test, BLOCK: Kohs Block Design Test, SYNUM: Synonyms Test, and INFO: Information Test.

**Table 3:** 

	Level 1	Level 2
Age		

Linear	*y*_*ij*_ = *π*_0*j*_ + *π*_1*j*_Age_*ij*_ + *r*_*ij*_	*π*_0*j*_ = *β*_00_ + *β*_10_(BaselineAge) + *β*_20_(BaselineTimeToDeath) + *U*_0*j*_
		*π*_1*j*_ = *β*_10_ + *β*_11_(BaselineAge) + *β*_21_(BaselineTimeToDeath) + *U*_1*j*_

Quadratic	*y*_*ij*_ = *π*_0*j*_ + *π*_1*j*_Age_*ij*_ + *π*_2*j*_Age_2*j*_^2^ + *r*_*ij*_	*π*_0*j*_ = *β*_00_ + *β*_10_(BaselineAge) + *β*_20_(BaselineTimeToDeath) + *U*_0*j*_
		*π*_1*j*_ = *β*_10_ + *β*_11_(BaselineAge) + *β*_21_(BaselineTimeToDeath) + *U*_1*j*_
		*π*_2*j*_ = *β*_20_ + *β*_21_(BaselineAge) + *β*_21_(BaselineTimeToDeath) + *U*_1*j*_

Time to death		

Linear	*y*_*ij*_ = *π*_0*j*_ + *π*_1*j*_TimeToDeath_*ij*_ + *r*_*ij*_	*π*_0*j*_ = *β*_00_ + *β*_10_(BaselineAge) + *β*_20_(BaselineTimeToDeath) + *U*_0*j*_
		*π*_1*j*_ = *β*_10_ + *β*_11_(BaselineAge) + *β*_21_(BaselineTimeToDeath) + *U*_1*j*_

Quadratic	*y*_*ij*_ = *π*_0*j*_ + *π*_1*j*_TimeToDeath_*ij*_ + *π*_2*j*_TimeToDeath_2*j*_^2^ + *r*_*ij*_	*π*_0*j*_ = *β*_00_ + *β*_10_(BaselineAge) + *β*_20_(BaselineTimeToDeath) + *U*_0*j*_
		*π*_1*j*_ = *β*_10_ + *β*_11_(BaselineAge) + *β*_21_(BaselineTimeToDeath) + *U*_1*j*_
		*π*_2*j*_ = *β*_20_ + *β*_21_(BaselineAge) + *β*_21_(BaselineTimeToDeath) + *U*_1*j*_

## Appendix

These models consist of two levels of analysis that allow researchers to explore how individuals change over time (Level 1) and how these changes vary across individuals (Level 2). Level 1 equation can be formulated as

(A.1) $$\begin{array}{c} y_{ij}=\pi_{0j}+\pi_{1j}{Time}_{ij}+r_{ij}, \end{array}$$

where *y*_*ij*_ represents the value of the outcome variable for individual *i* at time *j* (a function of time in the previous equation); *π*_0*j*_ represents a person *j* performance level when time is equal to 0 (intercept); and *π*_1*j*_ represents the rate of change for each unit of time (slope). In this equation, Time_*ij*_, is a metric of time over which change is structured.

Level 2 equations of an unconditional model can be formulated as

(A.2) π0j=β0+U0jπ1j=β1+U1j,

where *π*_0*j*_ and *π*_1*j*_ are considered as dependent variables, *β*_0_ and *β*_1_ being average level and rate of change, and *U*_0*j*_ and *U*_1*j*_ are each individual's deviation from the population average values, respectively. Specific equations for the present study are shown in Table 3.
